# Comparative evaluation of flavone from *Mucuna pruriens* and coumarin from *Ionidium suffruticosum* for hypolipidemic activity in rats fed with high Fat diet

**DOI:** 10.1186/1476-511X-11-126

**Published:** 2012-10-02

**Authors:** Satheesh Kumar Dharmarajan, Kottai Muthu Arumugam

**Affiliations:** 1 Department of Pharmacy, Annamalai University, Annamalai, Nagar, 608 002, India

**Keywords:** *M. pruriens*, *I.suffruticosum*, Flavone, Coumarin, Hyperlipidemia

## Abstract

The objective of the study is a comparative evaluation of flavone isolated from *Mucuna pruriens* and coumarin isolated from *Ionidium suffruticosum* was assessed for the hypolipidemic activity in rats fed with high fat diet. The acute toxicity study was found that flavone (*M.pruriens*) and coumarin (*I.suffruticosum*) are safe up to 100mg/kg, so one tenth of this dose (10mg/kg) was consider as a evaluation dose. High fat diet group of rats showed significant (p<0.001) elevation in plasma total and LDL-cholesterol, triglycerides and phospholipids. Administration of flavone (*M. pruriens*) and coumarin isolated from (*I.suffruticosum*) at the dose of 10mg/kg b.wt/day along with high fat diet significantly (p<0.001) prevented the rise in the plasma total and LDL-cholesterol, triglycerides and phospholipids than that of other extracts. However, treatment of coumarin isolated from (*I.suffruticosum*) had showed more cardio protective effect against hyperlipidemia than that of flavone (*M.pruriens*)*.*

## Introduction

Cardiovascular disease is a major problem worldwide. The World Health Organization estimates that this disease is responsible for the deaths of approximately 30,000 people each day [1]. Hyperlipidaemia is characterized by elevated serum total cholesterol, low-density lipoprotein cholesterol, very low density lipoprotein cholesterol and decreased high-density lipoprotein cholesterol levels [2]. As reported in literature synthetic drugs may be having serious side effects [3]. Statins may also be associated with some other rarely occurring side effects like nausea, abdominal pain, dyspepsia, diarrhoea or constipation and flatulence [4]. Medicinal plants, on the other hand, have been reported safer as compared topharmaceutically derived remedies [2].

*Mucuna pruriens* Linn belongs to the family fabaceae, traditionally in India the seeds of *Mucuna pruriens* are used as a tonic and aphrodisiac for male virility. It has been reported to be antidiabetic [5], analgesic and anti-inflammatory [6]. Its different preparations (from seeds) are used for the management of several free radical-mediated diseases such as ageing, rheumatoid arthritis, diabetes, atherosclerosis, male infertility and nervous disorders. *Ionidium suffruticosum* (Ging.) it belongs to the family Violaceae, it is widely used as traditional healers for the treatment of diseases like diabetes [7], male sterility [8], urinary tract infections and water retention [9]. Hence, the attempt is made for the comparative evaluation of flavone from *Mucuna pruriens* and Coumarin isolated from *Ionidium suffruticosum* for hypolipidemic activity in rats fed with high fat diet.

## Materials and methods

### Collection and identification of plant materials

The whole plant of *Mucuna pruriens* (Linn), were collected from Neiyur dam, Kanyakumari District of Tamil Nadu, India and the whole plant of *Ionidium suffruticosum* (Ging) were collected from Kilikulam, Tirunelveli District of Tamil Nadu, India. The taxonomic identification was made from Botanical Survey of Medicinal Plants Unit Siddha, Government of India, Palayamkottai. The whole plant of *Mucuna pruriens* (Linn) and *Ionidium suffruticosum* (Ging) were dried under shade, segregated, pulverized by a mechanical grinder and passed through a 40 mesh sieve. The powdered plant materials were stored in an airtight container.

### Chemicals

All the chemicals used in the study were of analytical grade, procured from the credible concerns e.g.: Sigma, Merck and Qualigens. Atorvastatin was provided as a generous gift sample by Ranbaxy Pharmaceuticals, India.

### Preparation of various extracts from *Mucuna pruriens* and *ionidium suffruticosum*

The whole plant of *Mucuna pruriens* (Linn) and *Ionidium suffruticosum* (Ging) were dried in shade and powdered. The powdered plant materials were successively extracted with petroleum ether (40-60°C) by hot continuous percolation method in Soxhlet apparatus [10] for 24 hrs. Then the marc was dried and then subjected to ethyl acetate (76-78°C) for 24 hrs, then marc was dried and then it was subjected to methanol (80°C) for 24 hrs. The solvent from the extracts was recovered under reduced pressure using rotary evaporator and subjected to freeze drying in a lyophilizer till dry powder was obtained.

### Isolation of flavone from methanol extract of *Mucuna pruriens*

The methanol extract of *Mucuna pruriens* was subjected to column chromatographic separation using normal phase silica gel column. The dark brown solid (20 g methanol extract of *Mucuna pruriens*) was adsorbed on silica gel (20 g) and transferred to a column of silica gel (200g equilibrated with benzene). Flavone (265mg) was eluted with ethyl acetate: methanol, 80:20 v/v from methanol extract of *Mucuna pruriens*.

### Isolation of coumarin from methanolic extract of *ionidium suffruticosum*

The methanolic extract of *Ionidium suffruticosum* was subjected to column chromatographic separation using normal phase silica gel column. The dark brown solid (20 g methanolic extract of *Ionidium suffruticosum*) was adsorbed on silica gel (20 g) and transferred to a column of silica gel (200g equilibrated with benzene). The Coumarin derivative (245 mg) was eluted with benzene: Chloroform 70:30, v/v from methanolic extract of *Ionidium suffruticosum*.

### Experimental animals

Healthy male Wistar rats (weighing 120-150g) were procured from Central Animal House, Rajah Muthiah Medical College, Annamalai University. The animals were kept in cages, 2 per cage, with relative humidity (55%) in a 12 hour light/dark cycle at 25±2°C. They were given access to water and a commercial diet *ad libitum*. The experiment were carried out as per the guidelines of Committee for the Purpose of Control and Supervision of Experiments on Animals (CPCSEA), New Delhi, India, and approved by the Institutional Animal Ethics Committee (IAEC), Annamalai University (Approved number: 160/1999/CPCSEA/745).

### Acute toxicity studies

Acute oral toxicity study was performed as per Organization and Economic Cooperation and Development (OECD) guidelines 423 adopted on 17^th^ December 2001 received from Committee for the purpose of Control and Supervision of Experimental Animals (CPCSEA). The rats were fed with flavone from *Mucuna pruriens* and Coumarin derivatives from *Ionidium suffruticosum* suspended in 1% gum acacia at the dose of 100mg/kg body weight. The animals were observed individually every 30 minutes after dosing the first 24hrs and thereafter daily for a total of 14 days. The time at which signs of toxicity appear and disappear was observed systematically and recorded for each animal.

### Experimental induction of hyperlipidemia

High fat diet was prepared by mixing Wheat flour 20.5%, roasted bengal gram 52.6%, skimmed milk powder 5%, casein 4%, refined oil 4%, coconut oil 9%, salt mixture with starch 4% and vitamin & choline mixture 0.5%, cholesterol 0.4% [11]. The diet was placed in the cage carefully and was administered for 14 days [12].

### Protocol for antihyperlipidemic activity

In the experiment a total number of 36 rats were used. The rats were divided into five groups of six each. Group I: control, Group II: High Fat Diet (HFD), Group III: HFD plus Flavone (*M. pruriens*) at the dose of 10mg/kg b.wt, Group IV: HFD plus Coumarin (*I. suffruticosum*) at the dose of 10mg/kg b.wt, Group V: HFD + standard drug atorvastatin (1.2 mg/kg b.wt). The drugs were suspended in 2% tween 80 [13] separately and fed to the respective rats by oral intubation. At the end of the study all the rats were sacrificed by cervical dislocation after overnight fasting. Just before sacrifice, blood was collected from the retro-orbital sinus plexus under mild ether anaesthesia and blood sample collected in heparinised tubes and plasma was separated. Liver, heart and aorta were cleared of adhering fat, weighed accurately and used for the preparation of homogenate. Animals were given enough care as per the Animal Ethical Committee’s recommendations.

### Biochemical analysis

Plasma samples were analyzed for total cholesterol, HDL-cholesterol and triglycerides using Boehringer Mannheim kits by Erba Smart Lab analyzer USA. LDL-cholesterol and VLDL-cholesterol were calculated by using Friedwald method [14]. Ester cholesterol [15] and free cholesterol [15] were analyzed by using digitonin. Portions of liver, heart and aorta tissues were blotted, weighed and homogenized with methanol (3 volumes) and the lipid extracts were obtained by the method of Folch et al*.* (1957) [16]. Extracts were used for the estimation of ester cholesterol and free cholesterol, triglycerides [17], and phospholipids [18]. The atherogenic Index was calculated by using the following formula.

(1) $$\text{Athorogenic Index}=\frac{Total\;cholesterol}{HDL}$$

### Statistical analysis

Results were expressed as mean ± SE of 6 rats in each group. The data were also analysed by one way analysis of variance (ANOVA) followed by Dunnet’s t-test. ‘P, value less than 0.05 is considered significant.

## Results and discussion

Balance between synthesis and degradation of biological tissues is maintained by Lipid metabolism. Development of hyperlipidemia disease is a complicated process involving accumulation of lipid containing particles in the walls of coronary arteries & other major arteries within the body. A high-fat diet causes cholesterol levels to increase in susceptible people, which leads to obesity.

The flavone isolated from *Mucuna pruriens* and coumarin derivatives isolated from *Ionidium suffruticosum* were found to be non-toxic upto the dose of 100mg/kg and did not cause any death of the tested animals. Therefore, one tenth of this dose (10mg/kg) was considered as the evaluation dose. As shown in Table 1, the weight gain in HFD group of rats significantly (p<0.001) higher than control rats reflecting the influence of high fat diet. The increment in the weight reduced significantly (p<0.001) by the administration of flavone from (*M.pruriens*) (Group III) and Coumarin (*I. suffruticosum*) (Group IV) at the dose of 10mg/kg as well as atorvastatin 1.2mg/kg in comparison with the HFD fed rats (Group II). Administration of Coumarin (*I. suffruticosum*) at the dose of 10mg/kg exhibited the significant reduction in bodyweight in comparison with the group received flavone (*M. pruriens*) (Group III). The weight reducing effect may be attributed to its potential to inhibit lipogenesis and enhanced thermogenesis, since obesity is associated with defective thermogenesis [19].

**Table 1:** Average Body weight changes in control and experimental rats

**Groups**	**Initial Weight (g)**	**Final Weight (g)**	**Average Body weight gain (g)**
Group I	137.34±0.47^bNS^	156.66±2.47^b*^	19.32±2.39^b*^
Group II	136.33±0.42^aNS^	198.64±2.58^a*^	62.31±1.90^a*^
Group III	136.71±0.41^aNS,bNS^	162.33±2.16^aNS,b**^	25.62±2.54^aNS, b*^
Group IV	136.57±0.53^aNS,bNS^	161.26±1.38^aNS,b*^	24.69±3.28^aNS,b*^
Group V	137.55±0.74^aNS,bNS^	161.51±1.74^aNS,b*^	23.96±3.39 ^aNS,b*^

Values are expressed as mean ± SE (n=6 rats); *P* values: *<0.001, **<0.05; NS: Non significant; a → group I compared with groups II, III, IV& V; b → group II compared with groups III, IV& V; Group I : Standard chow diet (Control); Group II : High Fat Diet (Negative Control); Group III : HFD + Flavone (*M.pruriens*) (10mg/kg b.wt); Group IV : HFD+Coumarin (*I. suffruticosum*) (10mg/kg b.wt); Group V : HFD + Atorvastatin (1.2 mg/kg b.wt) (Positive Control).

As shown in Table 2, HFD rats are showed significant increase (p<0.001) in plasma total cholesterol level as compared to control rats (group I). Earlier studies reveal significant elevation of lipid parameters in plasma and tissue response to atherogenic diet or high fat diet [20,21]. Treatment with flavone isolated from *Mucuna pruriens* and coumarin derivatives isolated from *Ionidium suffruticosum* at the dose of 10mg/kg body weight had showed a significant (p<0.001) decrease in the total cholesterol level compared to HFD rats (group II). Lowering high cholesterol levels significantly reduced the risk of heart attacks, strokes, and death. However, the rats received coumarin (*I. suffruticosum*) at the dose of 10mg/kg b.wt (Group IV) with HFD had showed restored the plasma cholesterol level near to normal as that of standard (Group V).

**Table 2:** **Effect of flavone from *Mucuna pruriens *and coumarin from *Ionidium suffruticosum *on plasma lipid profile in control and experimental rats**

**Groups**	**Total cholesterol (mg/dl)**	**Free cholesterol (mg/dl)**	**Ester cholesterol (mg/dl)**	**Phospholipid (mg/dl)**	**Triglyceride (mg/dl)**	**Athrogenic index**
Group I	69.81±0.51^b*^	23.67±0.43^b*^	46.14±0.61^b*^	155.87±0.24^b*^	78.57 ±0.20^b*^	2.90±0.08^b*^
Group II	167.17±0.26^a*^	49.41±0.12^a*^	117.76±0.57^a*^	208.33±0.33^a*^	121.83±0.33^a*^	9.73±0.21^a*^
Group III	77.43 ±0.29^a*,b*^	24.36±0.74^a*,b*^	53.07±0.82^a*,b*^	160.71±0.34^a*,b*^	84.03±0.40^a*,b*^	3.35±0.18^a*,b*^
Group IV	72.74±0.47^a*,b*^	23.05±0.40^a*,b*^	49.69±0.22^a*,b*^	156.47±0.12^a*,b*^	79.47±0.37^a*,b*^	2.88±0.84^a*,b*^
Group V	72.92 ±0.22^a*,b*^	23.44±0.55^a*,b*^	49.48±0.56 ^a*,b*^	156.29±0.28^a*,b*^	80.19 ±0.07^a*,b*^	2.97±0.53^a*,b*^

Values are expressed as mean ± SE (n=6 rats); *P* values: ^*^ < 0.001, ^**^ < 0.05; NS: Non Significant; a →group I compared with groups II, III, IV, V; b →group II compared with groups III, IV, V; Details of group I-V are same as in Table 1.

As shown in Tables 2, 3 and 4, the significant (P<0.001) increase in levels of both free and ester cholesterol were also observed in plasma and tissue of rats fed with high fat diet (group II) when compared to control rats (group I). This high cholesterol concentration in circulation may damage the endothelial cells lining the large arteries and aorta and this may be an initial event in the etiology of atherosclerosis [22]. Both plasma free and ester cholesterol reduced remarkably on treating the HFD rats with flavone (*M. pruriens*) and coumarin (*I. suffruticosum*) at the dose of 10mg/kg body weight*.* This lipid lowering effect may be due to the inhibition of hepatic cholesterogenesis or due to the increase in excretion of fecal sterol [23].

**Table 3:** **Effect of flavone from *Mucuna pruriens *and coumarin from *Ionidium suffruticosum *on tissues ester cholesterol profile in control and experimental rats**

**Groups**	**Ester cholesterol (mg/g tissue)**		
	**Liver**	**Heart**	**Aorta**
Group I	0.67 ± 0.02^b*^	2.86± 0.01^b*^	2.02±0.42^b*^
Group II	2.55±0.04^a*^	6.96±0.02^a*^	6.81±0.23^a*^
Group III	2.02±0.01^a*,b**^	3.78±0.03^a*^,^b*^	4.98±0.24^a*,b*^
Group IV	2.59±0.03 ^a*,b*^	3.10±0.01^a*,b*^	2.69±0.09^a*,b*^
Group V	1.84±0.01^a*,b*^	2.98±0.01^a*,b*^	2.83±0.11^a*,b*^

Values are expressed as mean ± SE (n=6 rats); *P* values: ^*^ < 0.001, ^**^ < 0.05; NS: Non Significant; a →group I compared with groups II, III, IV, V; b →group II compared with groups III, IV, V; Details of group I-V are same as in Table 1.

**Table 4:** **Effect of flavone from *Mucuna pruriens *and coumarin from *Ionidium suffruticosum *on tissues free cholesterol profile in control and experimental rats**

**Groups**	**Free cholesterol (mg/g tissue)**		
	**Liver**	**Heart**	**Aorta**
Group I	0.65±0.01^b*^	0.69± 0.01^b*^	0.40±0.01^b*^
Group II	1.24±0.01^a**^	1.17±0.03 ^a*^	1.86±0.02^a*^
Group III	0.89 ±0.01^a*,b**^	0.12±0.01^a**,b*^	0.73±0.01^a*,b*^
Group IV	0.73±0.01^a*,b*^	0.66±0.01^a*,b*^	0.62±0.01^a*,b*^
Group V	0.81±0.04^a*,b*^	0.64±0.04^a*,b*^	0.63±0.04^a*,b*^

Values are expressed as mean ± SE (n=6 rats); *P* values: ^*^ < 0.001, ^**^ < 0.05; NS: Non Significant; a →group I compared with groups II, III, IV, V; b →group II compared with groups III, IV, V; Details of group I-V are same as in Table 1.

As shown in Tables 2 and 5. The concentration of plasma and tissue triglyceride elevated in rats fed with high fat diet (group II) as compared to control rats (group I). HFD rats had significant increase in the level of plasma triglyceride due to decrease in the activity of lipoprotein lipase [24,25]. Both plasma and tissue triglyceride levels were significantly reduced in rats treated with flavone (*M. pruriens*) at the dose of 10mg/kg body weight, coumarin (*I. suffruticosum*) at the dose of 10mg/kg body weight and as well as standard drug atorvastatin along with HFD when compared with rats fed with high fat diet (group II). Administration of coumarin (*I. suffruticosum*) at the dose of 10mg/kg body weight reduced the triglyceride level significantly (p<0.001) in comparison with flavone (*M. pruriens*). The flavone (*M. pruriens*) and coumarin (*I. suffruticosum*) may have stimulation of lipoprotein lipase activities resulting in decrease of plasma triglyceride and might increase the uptake of triglyceride from plasma by skeletal muscle and adipose tissues [26].

**Table 5:** **Effect of flavone from *Mucuna pruriens *and coumarin from *Ionidium suffruticosum *on tissues Triglyceride level in control and experimental rats**

**Groups**	**Triglyceride (mg/g tissue)**		
	**Liver**	**Heart**	**Aorta**
Group I	7.95±0.01^b*^	9.17±0.01^b*^	9.48±0.33^b*^
Group II	18.11±0.19^a*^	28.79±0.16^a*^	19.37±0.78^a*^
Group III	9.57±0.15^a*, b*^	16.78± 0.21^a**,b*^	12.67±0.11^a*,b*^
Group IV	8.24±0.10^a*,b*^	15.47 ± 0.12^a*,b*^	12.08 ± 0.12^a*,b*^
Group V	8.31±0.69^a*,b*^	15.21±0.36^a*,b*^	12.15 ± 0.01^a*,b*^

Values are expressed as mean ± SE (n=6 rats); *P* values: ^*^ < 0.001, ^**^ < 0.05; NS: Non Significant; a →group I compared with groups II, III, IV, V; b →group II compared with groups III, IV, V; Details of group I-V are same as in Table 1.

As shown in Tables 2 and 6, the concentration of plasma and tissue phospholipids significantly increased in rats fed HFD (group II) as compared to control animals (group I). This may be due to decreased phospolipase activity [27]. (Group II). Both plasma and tissue phospholipids levels significantly decreased in rats treated with flavone (*M. pruriens*) 10mg/kg body weight and coumarin (*I. suffruticosum*) 10mg/kg body weight and as well as standard drug atorvastatin along with HFD when compared with rats fed with high fat diet (group II). Whereas, the rats received coumarin (*I. suffruticosum*) at the dose of 10mg/kg body weight (Group IV) exhibited significant reduction in triglyceride level in comparison with flavone (*M. pruriens*) (Group III). The reduced concentration of phospholipids may also be due to the enhanced activity of phospholipases [11].

**Table 6:** **Effect of flavone from *Mucuna pruriens *and coumarin from *Ionidium suffruticosum *on tissues Phospholipids level in control and experimental rats**

**Groups**	**Phospholipids (mg/g tissue)**		
	**Liver**	**Heart**	**Aorta**
Group I	12.37±0.45^b*^	18.04 ±0.67^b*^	10.32±0.05^b*^
Group II	25.14±0.69^a*^	26.47±0.10^a*^	17.45± 0.29^a*^
Group III	17.81 ± 0.01^a*,b*^	20.14±0.65^a*,b*^	13.06± 0.13^a*,b**^
Group IV	16.45±0.06^a*,b*^	19.45±0.73^a*,b*^	12.47±0.12^a*,b*^
Group V	17.45±0.77^a*,b*^	19.08±0.18^a*,b**^	12.02± 0.10^a*,b*^

Values are expressed as mean ± SE (n=6 rats); *P* values: ^*^ < 0.001, ^**^ < 0.05; NS: Non Significant; a →group I compared with groups II, III, IV, V; b →group II compared with groups III, IV, V; Details of group I-V are same as in Table 1.

Atherogenic Index (AI) indicates the deposition of foam cells or plaque or fatty infiltration or lipids in heart, coronaries, aorta, liver and kidney. The higher the AI, higher is the risk of above organs for oxidative damage. As shown in Table 2, the atherogenic index (TG/HDL-C ratio) used to predict risk of CHD and marker of small, dense LDL-C (an atherogenic lipoprotein) [28,29] were significantly reduced by the flavone from *M.pruriens* and coumarin from *I.suffruticosum*, indicating the beneficial effect of flavone and coumarin in cardiovascular diseases.

As shown in Table 7, the reduction in the HDL produced by the group of animals fed with HFD was significant (P<0.001) in comparison with group I animals. This result is highly significant in that low HDL-cholesterol is now considered as the most significant risk factor for atherosclerosis [30] (Brewer, 2004). However, the treatment with flavone isolated from *Mucuna pruriens* (Group III) and coumarin derivatives isolated from *Ionidium suffruticosum* (Group IV) had significantly increased the HDL-cholesterol level when compared to HFD rats (Group II). It has clearly been demonstrated that a relationship exists between increased concentration of HDL-C and decreased morbidity-and mortality-rate in cardiovascular patients [31].

**Table 7:** **Effect of flavone from *Mucuna pruriens *and coumarin from *Ionidium suffruticosum *on plasma lipoprotein in control and experimental rats**

**Groups**	**HDL cholesterol (mg/dl)**	**LDL cholesterol (mg/dl)**	**VLDL cholesterol (mg/dl)**
Group I	24.04±0.16^b*^	30.06±0.41^b*^	15.71±0.10^b*^
Group II	17.17±0.22^a*^	125.64±0.55^a*^	24.36±0.17^a*^
Group III	23.11±0.12^a*,b*^	37.52± 0.37^a*,b*^	16.80±0.20^a*,b **^
Group IV	25.27±0.19^a*,b*^	31.58± 0.08^a*,b*^	15.89±0.18 ^a**,b*^
Group V	24.51±0.26^a*, b*^	32.37± 0.19^a*,b*^	16.04±0.03^a*,b*^

Values are expressed as mean ± SE (n=6 rats); *P* values: ^*^ < 0.001, ^**^ < 0.05; NS: Non Significant; a →group I compared with groups II, III, IV, V; b →group II compared with groups III, IV, V; Details of group I-V are same as in Table 1.

As shown in Table 7, the elevated levels of LDL and VLDL-cholesterol in rats fed with HFD (group II) was significant (P<0.001) in comparison with control rats (group I). High cholesterol diet increased serum cholesterol and LDL-C level significantly. Clinical and epidemiological studies have proved that individuals with elevated LDL show an increased risk for cardiovascular diseases [32]. Treatment of flavone from *M.pruriens* (Group III) and coumarin from *I.suffruticosum* (Group IV) markedly reduced the level of plasma LDL-cholesterol and VLDL-cholesterol when compared to HFD rats (group II). In comparison of flavone from *Mucuna pruriens* (Group III) and coumarin from *Ionidium suffruticosum* (Group IV) with HFD (Group II) rats, the coumarin from *Ionidium suffruticosum* (Group IV) was showed significant reduction on both LDL-cholesterol and VLDL-cholesterol than that of group III rats. Recent research has revealed that a 4-5% decrease in LDL-cholesterol results in a 5-10% decrease in the occurrence of coronary heart disease (CHD) [33].

## Conclusion

On the basis of the present investigation was observed as the Coumarin derivatives isolated from *Ionidium suffruticosum* had showed a better antihyperlipidemic activity in comparison with flavone isolated from *Mucuna pruriens.* The Coumarin derivatives isolated from *Ionidium suffruticosum* is beneficial in preventing hypercholesterolemic atherosclerosis and reducing risk factors for coronary artery disease.

## Competing interests

No disclosures. There is no affiliation, financial agreement or any other involvement with any company.

## Authors’ contributions

DSK carried out the in vivo studies, designed the protocol of the study and drafted the manuscript. AKM participated in design and coordination of the study. All authors read and approved the final manuscript.
